# 3D‐printed educational model for anatomy‐driven preparation and retraction cord placement

**DOI:** 10.1002/jdd.13819

**Published:** 2025-01-06

**Authors:** Alexey Unkovskiy, Franziska Schmidt, Florian Beuer, Jeremias Hey

**Affiliations:** ^1^ Department of Prosthodontics Geriatric Dentistry, and Craniomandibular Disorders Charité‐Universitätsmedizin Berlin, Corporate Member of Freie Universität Berlin, Humboldt‐Universität zu Berlin Berlin Germany; ^2^ Department of Dental Surgery Sechenov First Moscow State Medical University Moscow Russia; ^3^ Department of Prosthodontics Martin Luther University Halle‐Wittenberg Halle Germany

**Keywords:** prosthodontics, clinical skills/topics, skills/doctoring, clinical skills/topics, prosthodontics, dental specialties & sub‐specialties, advanced education general dentistry, education, educational technology, education

## 1 PROBLEM

For decades the standard typodont training models by KaVo or Frasaco have been used for dental education. The main criticism of these models relates to the simplistic and reduced imitation of dentition and morphology of the oral cavity; therefore, their educational value is moderate. In 2021, the curriculum and licensing regulations were replaced by new statutes in Germany with a main focus being put on clinical practice instead of preclinical courses. This aspect required more detailed training models that can realistically reproduce the whole dental, bone and soft tissue anatomy allowing for more realistic training. However, this issue can be regarded as an international challenge. In recent times some new education models were introduced for practicing endodontic skills.^1^, ^2^ The others concentrated on exercising surgical skills and implantology.^3^, ^4^ For educating dental students in crown preparation a group of authors concentrated on enamel, dentin core, and pulp segmentation.^5^ However, all these models lack a sufficient periodontal anatomy which is also crucial for soft tissue management, for example during the gingiva retraction for impression taking.

## 2 SOLUTION

**FIGURE 1:**
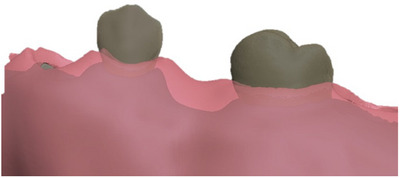
Design of the educational model with adjusted supracrestal dimension. The design of the bony alveolar bone, teeth, and gingival mask was exported separately.

For the 3D data acquisition, preexisting cone beam computer tomography data were uploaded into an artificial intelligence software (Diagnocat). The maxilla and upper dentition were segmented and exported as a separate file in surface tessellation language. The tooth anatomy was separated into dentin core and enamel cap. The gingival mask was designed based on the intraoral scan using Trio4 (3shape) using free‐form software (Zbrush, Pixologic). Care was taken to keep the biologically determined thickness of the gingiva and the supracrestal dimension of each tooth. All parts of the designed model were exported separately (Figure 1).

## 3 RESULTS

The designed model was 3D‐printed using the Polyjet method (Stratasys J5 Digital Anatomy). This allowed for a multicolor, multimaterial 3D‐printing with TissueMatrix (MED410), GelMatrix (FLG111), and BoneMatrix (RGD526) materials. The bony and dental structures were printed using hard material, and the gingiva was printed simultaneously using soft ones.

**FIGURE 2:**
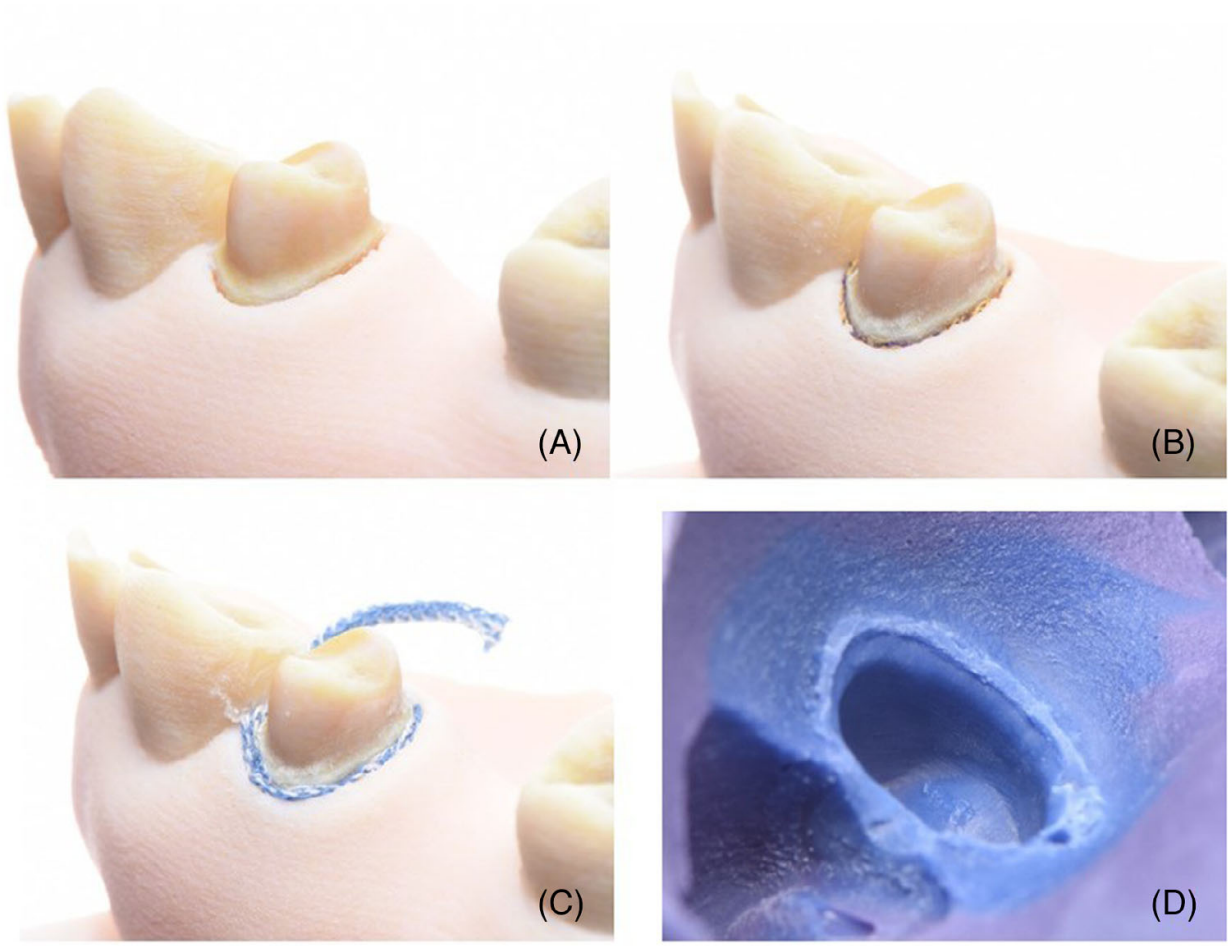
Cord placement using double‐cord technique. (A) Prepared tooth ready for retraction; (B) the first cord for the vertical retraction; (C) the second cord for the horizontal retraction; (D) the final impression with the displayed preparation margin.

During tooth preparation, the distinction between enamel and dentine was clear. This allowed for the control of preparation invasiveness (Figure 2A). An antagonist model for occlusal clearance can be printed in a single material using a less expensive 3D printing method. For the gingival retraction, the double‐cord technique was used. The first retraction cord was placed dry in the sulcus (Figure 2B). It was completely submerged in the sulcus, as the supracrestal position was sufficient. Afterward, the second cord was placed dry for horizontal retraction (Figure 2C). The realistic feeling and soft tissue resistance were achieved due to the accurate supracrestal dimensions in this educational model, unlike the typodont model (Figure 2D). The soft tissue was removed to almost the same position after the cord was extracted.
